# Optimising prednisolone or prednisone replacement in adrenal insufficiency

**DOI:** 10.1530/EC-23-0097

**Published:** 2023-07-26

**Authors:** Angelica Sharma, Katharine Lazarus, Deborah Papadopoulou, Hemanth Prabhudev, Tricia Tan, Karim Meeran, Sirazum Choudhury

**Affiliations:** 1 Division of Diabetes, Endocrinology and Metabolism, Department of Metabolism, Digestion and Reproduction, Imperial College London, London, UK; 2 Department of Endocrinology, Imperial College Healthcare NHS Trust, London, UK; 3 Department of Clinical Biochemistry, North West London Pathology, London, UK

**Keywords:** prednisolone, prednisone, pituitary, adrenal, glucocorticoids

## Abstract

**Context:**

Patients with adrenal insufficiency (AI) have a higher mortality than the general population, possibly because of excess glucocorticoid exposure at inappropriate times. The cortisol circadian rhythm is difficult to mimic with twice- or thrice-daily hydrocortisone. Prednisolone is a once-daily alternative which may improve patient compliance through its convenience.

**Objectives:**

Prednisolone day curves can be used to accurately downtitrate patients to the minimum effective dose. This study aimed to review prednisolone day curves and determine therapeutic ranges at different time points after administration.

**Methods:**

Between August 2013 and May 2021, 108 prednisolone day curves from 76 individuals receiving prednisolone replacement were analysed. Prednisolone concentrations were determined by ultra-high-performance liquid chromatography-tandem mass spectrometry. Spearman’s correlation coefficient was used to determine the relationship between 2-, 4-, and 6-h prednisolone levels compared to the previously validated standard 8-h prednisolone level (15–25 μg/L).

**Results:**

The median dose was 4 mg of prednisolone once daily. There was a strong correlation between the 4- and 8-h (*R* = 0.8829, *P* ≤ 0.0001) and 6- and 8-h prednisolone levels (*R* = 0.9530, *P* ≤ 0.0001). Target ranges for prednisolone were 37–62 μg/L at 4 h, 24–39 μg/L at 6 h, and 15–25 μg/L at 8 h. Prednisolone doses were successfully reduced in 21 individuals, and of these, 3 were reduced to 2 mg once daily. All patients were well upon follow-up.

**Conclusion:**

This is the largest evaluation of oral prednisolone pharmacokinetics in humans. Low-dose prednisolone of 2–4 mg is safe and effective in most patients with AI. Doses can be titrated with either 4-, 6-, or 8-h single time point drug levels.

## Introduction

Patients with adrenal insufficiency (AI) have premature morbidity and mortality (1). Mildly elevated levels of glucocorticoids or non-circadian timing of therapy may contribute towards cardiovascular disease and increased mortality (2).

Replacement with glucocorticoid therapy is the mainstay of treatment, with an objective to mimic the circadian cortisol profile (3). Current guidelines recommend the use of hydrocortisone in divided doses or low-dose (3–5 mg) prednisolone (4).

Over-replacement with glucocorticoids avoids adrenal crisis at the expense of an increased risk of developing multiple comorbidities, including obesity, diabetes, cardiovascular disease, and osteoporosis (5). It is crucial to achieve an optimum dose for symptomatic control and avoidance of adrenal crises whilst minimising overexposure to glucocorticoids (6). This may be particularly challenging due to the inter-individual variability in physiological response and metabolic clearance of exogenous glucocorticoids (4).

Traditionally used hydrocortisone therapy has a short half-life of approximately 1.8 h and, therefore, requires multiple doses to maintain therapeutic levels (7). The use of multiple-dose glucocorticoids results in a non-physiological profile with supraphysiological levels in the evenings when individuals are more sensitive to the effects of cortisol (8).

In comparison, prednisolone has a longer half-life attributed to the presence of a double bond between C1 and C2, thus allowing for once-daily regimens. In a study of patients with congenital adrenal hyperplasia, prednisolone was six to eight times more potent than hydrocortisone (6, 9). Consequently, a hydrocortisone dose of 20 mg daily (e.g. 10 mg + 5 mg + 5 mg) is equivalent to a prednisolone dose of 2–4 mg once daily. This may be because prednisolone has half the affinity for corticosteroid-binding globulin as compared to cortisol, and therefore higher concentrations of unbound prednisolone are present (10). Furthermore, prednisolone has a longer half-life and greater avidity for the glucocorticoid receptor and takes a longer time to dissociate. Once-daily prednisolone regimens are associated with greater patient satisfaction and compliance (5).

Concerns about adverse metabolic outcomes associated with prednisolone are based on evidence using higher doses of prednisolone. A study analysing 25 patients receiving either hydrocortisone (30 mg/day) or prednisolone (7.5 mg/day) concluded that a greater proportion of patients treated with prednisolone developed densitometric osteoporosis (11). Data from European Adrenal Insufficiency Registry compared cardio-metabolic profiles between individuals receiving prednisolone (average dose 5 mg) vs hydrocortisone (average dose 21.5 mg) (10). In this cohort, those receiving prednisolone had significantly higher total cholesterol and low-density lipoprotein levels compared with those receiving hydrocortisone. A large population-based study that reviewed historical GP prescriptions for prednisolone and hydrocortisone found that patients who had been on prednisolone had a higher mortality than those who had been on hydrocortisone, although the doses used were not reported (12). The excess mortality may be the result of over-replacement with prednisolone, as higher doses such as 5 mg in the morning and 2.5 mg in the evening were commonly used. These findings have not been reproducible in subsequent studies with lower doses of prednisolone, where results have demonstrated no significant difference in parameters, including bone density, HbA1C, blood pressure, body mass index, and waist circumference, between prednisolone and hydrocortisone use (5, 10, 13, 14).

There remains a paucity of evidence on the optimum prednisolone dose to ensure adequate replacement whilst minimising adverse side effects. If 15–25 mg hydrocortisone is the correct replacement dose, then prednisolone doses of 1.8–4.2 mg daily may be more appropriate (13).

At Imperial College Healthcare NHS Trust (ICHNT), an 8-h prednisolone level between 15 and 25 μg/L indicates adequate replacement (13, 15). However, the 8-h time point may be inconvenient for patients attending outpatient clinics, and therapeutic levels at other time points are important. The use of prednisolone levels may also have wider applicability in guiding prednisolone dosing in patients who may eventually be weaned off prednisolone completely.

To our knowledge, this is the largest evaluation of prednisolone pharmacokinetics in patients with AI. Previous studies have evaluated prednisolone pharmacokinetics in healthy volunteers at larger doses but have been limited by sample size (16, 17).

## Materials and methods

### Design

We analysed individuals receiving established prednisolone therapy who had prednisolone day curves performed between August 2013 and May 2021 at ICHNT. Prednisolone day curves were performed as part of routine clinical care and dose optimisation. Between 2013 and 2015, prednisolone day curves were used to optimise the prednisolone assay. Day curve results were later validated against 8-h prednisolone levels by 2015 (13), and subsequently, patients were routinely managed using 8-h levels for their convenience. Thereafter, day curves were performed based on clinical assessment and in challenging cases.

Demographic data, cause of adrenal failure (primary, secondary, or glucocorticoid-induced), laboratory values, and clinical measures were obtained from interrogating electronic medical records. All patients with secondary adrenal insufficiency (SAI) were assessed 6 weeks following pituitary surgery with a dynamic pituitary function test to assess their requirement for replacement glucocorticoid therapy.

We included individuals in both outpatient and inpatient settings who were receiving prednisolone replacement for more than 6 months. The prednisolone dose was administered in the early morning, in the fasted state.

Pharmacokinetic data were derived using the values obtained from prednisolone day curves generated from a total of 76 individuals. One patient (patient D) who was on prednisone 10 mg once daily from the United States for a chronic autoimmune disease wanted to convert to prednisolone as he moved to the UK and requested day curves to compare them.

As the samples in this study were collected as part of routine clinical care and were part of a subsequent audit (registration number: END_021), informed consent was not required.

### Measurement of prednisolone levels

Serum samples were obtained to measure prednisolone levels up to 8 h following administration of an individual’s regular replacement prednisolone dose.

Blood samples were collected into SST BD Vacutainer tubes, containing a serum clot activator and a serum separating gel. All specimens were spun and separated within 4 h, and serum was stored at a temperature of −20°C before analysis.

### Analytical methods

Prednisolone concentrations were determined by ultra-high-performance liquid chromatography tandem mass spectrometry (Waters Quattro Premier Mass Spectrometer). The assay is controlled using custom internal quality controls and is a UKAS-accredited assay. This method has demonstrated intra-assay and inter-assay coefficient variations of 2.7 and 4.1%, respectively, at a prednisolone concentration of 50 μg/L. In-depth methodology is detailed in a previous paper by Williams *et al.* (13). Time points within a single-day curve for each individual were quantified on one assay run.

### Data analysis and statistics

The mean and standard deviation (±s.d.) were calculated for parametric data. The median and interquartile range (IQR) were used to describe non-parametric continuous data. Categorical variables were described using proportions and frequencies. The Spearman’s rank correlation was used to determine the strength and direction of relationship between 8-h prednisolone levels vs 2-, 4-, and 6-h levels.

Day curve data from patients receiving 2–6 mg were used to generate a semi-log plot. The 95% confidence intervals of the slope of the graph were determined. The modulus of these values was taken as the elimination constant (*k*). Using the first-order elimination equation, where (*t*) is time, *C*_0_ is the initial concentration, and *C*_(*t*)_ is the concentration at (*t*):


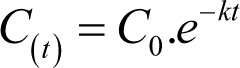



The existing 15–25 μg/L 8-h target range was used to generate target ranges at 4 and 6 h.

Measured prednisolone concentrations were also plotted against time to produce prednisolone day curves. The peak level (*C*_max_) and time to peak level (*T*_max_) were determined using actual collection time points. The area under the prednisolone level-time curve (AUC) was determined using the trapezoid method.

Statistical significance was defined as a *P*-value **<**0.05. All graphs, areas under the curve to 24 h (AUC_0–24h_), and pharmacokinetic data were created using GraphPad Prism 6 (GraphPad Software).

## Results

### Baseline characteristics

**Figure 1:**
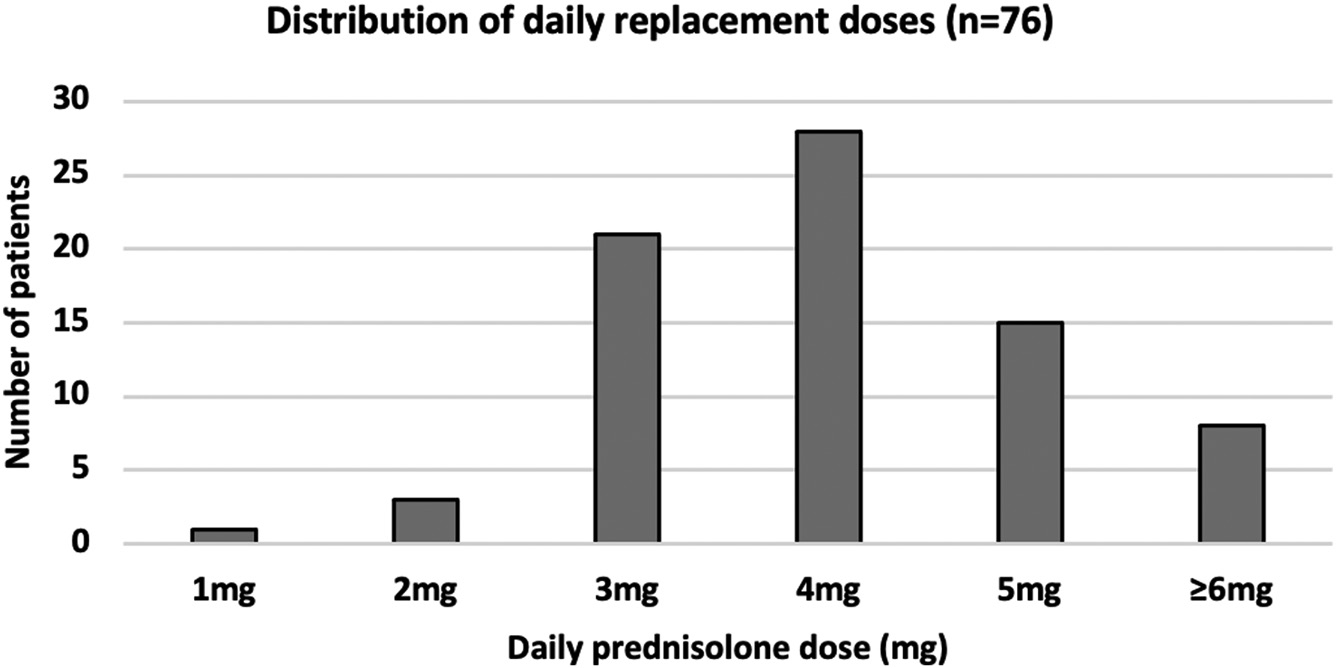
Distribution of daily replacement doses that individuals (*n* = 76) received.

In total, 108 prednisolone day curves were analysed from 76 individuals. Baseline characteristics are summarised in Table 1. Figure 1 illustrates the distribution of daily replacement doses that individuals received in this cohort.

**Table 1:** Baseline characteristics.

Parameter	Patient population (*n = *76)
Age, years (mean (s.d.))	61 (±13)
Gender (female)	60.5% (*n = *46)
Reason for prednisolone administration	
Primary adrenal insufficiency (PAI)	7.9% (*n = *6)
Secondary adrenal insufficiency (SAI)	63.2% (*n = *48)
Glucocorticoid-induced AI	28.9% (*n = *22)
Median (IQR) dose of prednisolone, mg	4.0 (3.0–5.0)
Median (IQR) dose of fludrocortisone, μg (PAI, *n = *6)	100 (50–125)

Within our patient cohort, 7.9% (*n* = 6) had primary AI (PAI), 63.2% (*n* = 48) had secondary AI (SAI), and 28.9% (*n* = 22) had tertiary AI (glucocorticoid-induced AI) (Table 1). The causes of SAI are summarised in Table 2.

**Table 2:** Causes of secondary adrenal insufficiency.

Cause of secondary adrenal insufficiency	Patient population (*n = *48)
Pathology requiring trans-sphenoidal pituitary surgery	
Cushing’s disease	16.6% (*n = *8)
Pituitary adenoma	37.5% (*n = *18)
Craniopharyngioma	12.5% (*n = *6)
Pituitary apoplexy	6.2% (*n = *3)
Rathke cleft cyst	2.1% (*n = *1)
Unilateral adrenalectomy	
Cushing’s syndrome	10.4% (*n = *5)
Pheochromocytoma (with ACTH co-secretion)	4.2% (*n = *2)
Empty sella syndrome	2.1% (*n = *1)
Sheehan’s syndrome	2.1% (*n = *1)
Langerhans’ cell histiocytosis	2.1% (*n = *1)
Lymphocytic hypophysitis	2.1% (*n = *1)
Hypoxic brain injury (cortisol deficiency)	2.1% (*n = *1)

### Pharmacokinetics

In the total population, the median (IQR) dose of prednisolone was 4 (3–5) mg corresponding to a mean (±s.d.) maximal time to peak level (*T*_max_) of 1.7 (±1) h after administration, with a median (IQR) 8-h level of 29 (19– 42.7) μg/L. The median (IQR) half-life was 3.1 (2.6–3.6) h with an AUC of 485 (366.5–703.9) μg · h/L. The median half-life of prednisolone remained similar at each prednisolone dose analysed.

Pharmacokinetic data are summarised in Table 3. Individuals are routinely commenced on a standard dose of 4 mg, many of whom are yet to be downtitrated. The individuals receiving 3 mg or less (Table 3) have had their doses actively reduced using the prednisolone levels obtained from day curves. This accounts for the 8-h concentrations within the target range for individuals receiving <2 and 3 mg but being above 25 μg/L in the other groups.

**Table 3:** Pharmacokinetic data of total population and by prednisolone dose.

Pharmacokinetics	Total (*n* = 76)	≤2 mg (*n* = 4)	3 mg (*n* = 21)	4 mg (*n* = 28)	5 mg (*n* = 15)	≥6 mg (*n* = 8)
C 8 h, μg/L (median (IQR))	29 (19–42.7)	22.2 (16.8–23.1)	21.4 (16–36)	29 (19.4–39.5)	37.1 (26.8–44.8)	48.5 (27.3–93.3)
AUC, μg h/L (median (IQR))	485 (366.5–703.9)	328 (229.1–387.7)	405 (361–525.5)	449.6 (424–681.9)	487 (329.3–775.2)	693.9 (458.6–1237.9)
*T*_max_, h (mean (s.d.))	1.7 (1)	1.6 (0.5)	1.7 (0.6)	2 (0.8)	2.3 (1.4)	1.9 (1.4)
Half-life, h (median (IQR))	3.1 (2.6–3.6)	3 (2.9–3.1)	3 (2.4–3.6)	3.1 (2.7–3.5)	3.1 (2.7–4)	3.4 (2.5–4.3)

### Prednisolone day curves

**Figure 2:**
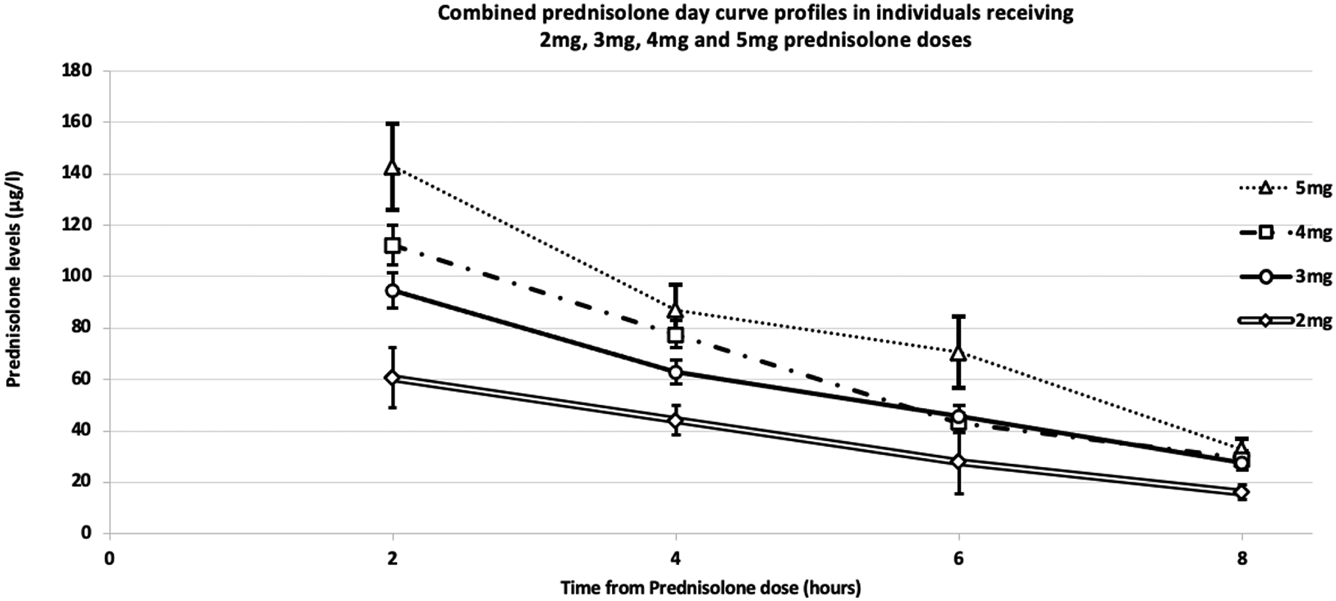
Combined prednisolone day curve profiles in individuals receiving 2 mg (*n* = 2), 3 mg (*n* = 21), 4 mg (*n* = 28), and 5 mg (*n* = 15) prednisolone doses. Error bars represent the standard error of the mean (s.e.m.).

Figure 2 summarises prednisolone day curves for individuals on doses 3 mg (*n* = 21), 4 mg (*n* = 28), and 5 mg (*n* = 15) using average prednisolone levels. These levels were used to slowly downtitrate the individual doses.

### Multiple dose profiles

**Figure 3:**
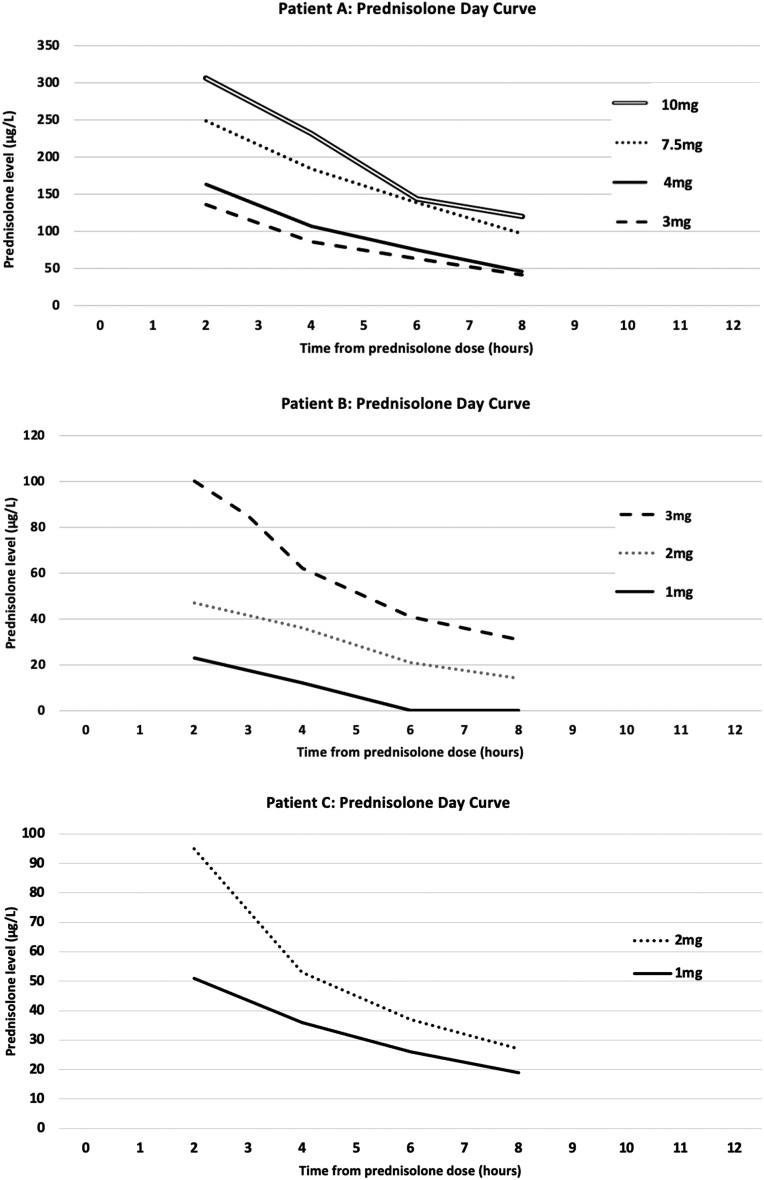
(A) Prednisolone day curves for patient A at 3, 4, 7.5, and 10 mg prednisolone doses. (B) Prednisolone day curves for patient B at 1, 2, and 3 mg prednisolone doses. (C) Prednisolone day curves for patient C at 1 and 2 mg prednisolone doses.

Of the 108 prednisolone day curves analysed, 22 individuals had repeat day curves on different doses of prednisolone. Figure(s) 3A, B, and C demonstrate the metabolism of varying dosages of prednisolone over a period of 8 h in three individuals. Patient A (Fig. 3A) had a bilateral adrenalectomy many years previously for recurrent pituitary dependent Cushing’s disease. She was gradually weaned from a prednisolone dose of 10 mg (8-h level: 120.2 μg/L) to a dose of 3 mg (8-h level: 37 μg/L) and finally to 2 mg (8-h level: 21 μg/L) which she has remained well on for several years. Patient B (Fig. 3B) was weaned from a dose of 3 mg to 1 mg – however, on a dose of 1 mg, the 8-h level was undetectable, and the patient felt unwell and hence a maintenance dose of 2 mg was established with an 8-h level of 16 μg/L. Patient C (Fig. 3C) was weaned from a prednisolone dose of 2 to 1 mg, with an 8-h level of 19 μg/L and a 6-h level of 36 μg/L. After this dose reduction, the baseline cortisol increased to 194 mmol/L, and she was weaned off prednisolone and remains clinically well.

### Prednisolone vs prednisone

For centres using prednisone, Table 4 summarises serum prednisolone levels after administration of prednisone (United States Pharmacopeia) and prednisolone (British Pharmacopeia). An oral dose of 10 mg of both medications was taken on consecutive days by patient D. After oral intake, prednisone and prednisolone (10 mg) were rapidly absorbed and identical serum concentrations of 161 μg/L were obtained at 90 min post ingestion. The 8-h level was 37 μg/L following prednisone and 35 μg/L following prednisolone.

**Table 4:** Prednisolone levels (μg/L) in patient D after an oral dose of prednisone and prednisolone on consecutive days.

Time (min)	10 mg prednisone	10 mg prednisolone
75	166	170
90	161	161
105	154	155
120	146	146
240	109	98
360	64	64
480	37	35
600	18	22

### Correlation of prednisolone levels and time of measurement

**Figure 4:**
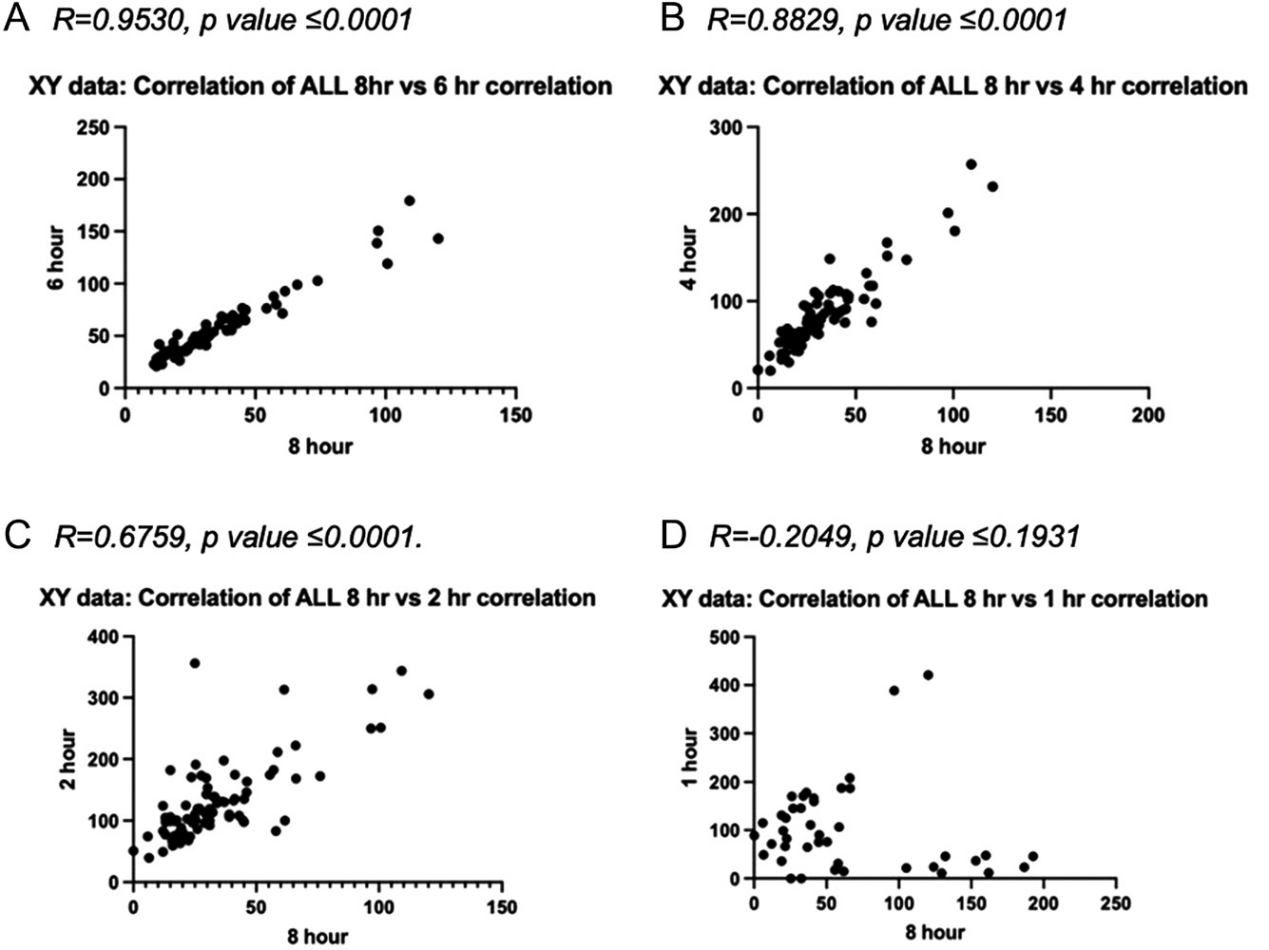
(A–D): Correlations between (A) 8- vs 6-h prednisolone levels; (B) 8- vs 4-h prednisolone levels; (C) 8- vs 2-h prednisolone levels; and (D) 8- vs 1-h prednisolone levels.

Figure 4 demonstrates the correlation between 8-h prednisolone levels as compared to 6-, 4-, 2-, and 1-h prednisolone levels.

There was a strong correlation between 8- and 6-h prednisolone levels (*R* = 0.9530, *P* ≤ 0.0001) and 8- and 4-h prednisolone levels (*R* = 0.8829, *P* ≤ 0.0001).

### Determining target ranges

**Figure 5:**
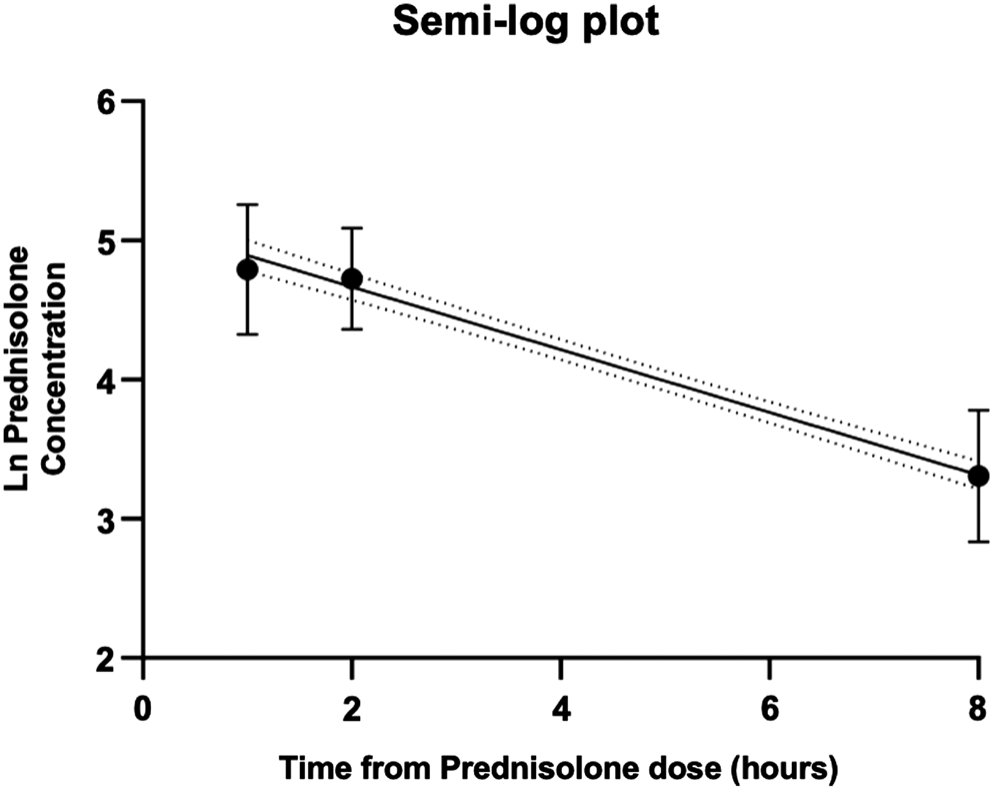
Semi-log plot.

**Figure 6:**
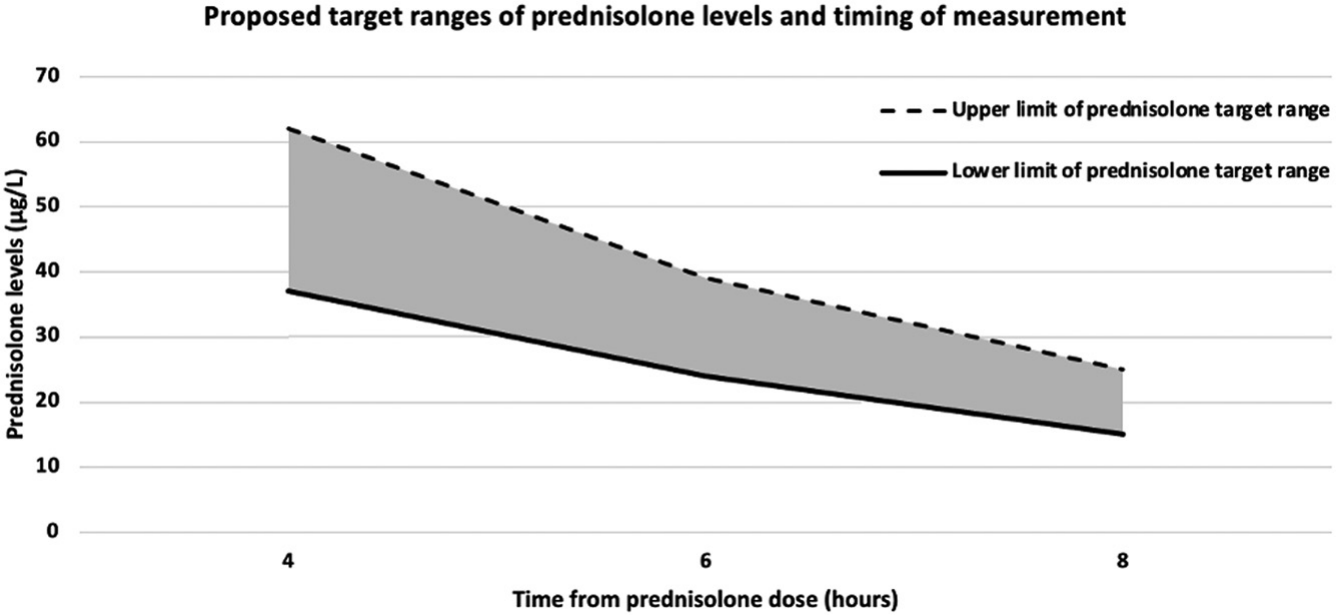
Proposed target ranges of prednisolone levels and timing of measurement.

Target ranges were determined using a semi-log plot (Table 5; Figs. 5 and 6). The calculated half-life was 3.1 h (95% CI: 2.80–3.41) based on an elimination constant of 0.23 h^−1^.

**Table 5:** Prednisolone level target ranges (μg/L) at 4- and 6-h time points.

Time from prednisolone dose (*n* = number of values)	Target range (μg/L)
6 h (*n* = 76)	24–39
4 h (*n* = 76)	37–62

### Clinical outcomes

Previous studies have stated that replacement is appropriate if the 8-h level of prednisolone is between 15 and 25 μg/L. In our patient population, 55.2% (*n* = 42/76) of individuals had prednisolone levels at 8 h above the optimum range and could have their dose reduced.

To determine if patients reported adverse symptoms whilst on prednisolone replacement, electronic medical records from follow-up appointments were reviewed. The last day curve reviewed was completed in May 2021, and the final review of electronic patient records began in July 2022. This ensured a minimum of 1-year follow-up for all individuals in the dataset. The decision to wean prednisolone was made by a panel of endocrinologists in a multi-disciplinary team meeting. Individuals were deemed ‘well’ if they remained asymptomatic, with an adequate blood pressure and normal electrolytes on a replacement dose of prednisolone. Of the 63 (82.9%) who were deemed well, 35 (55.6%) had an 8-h prednisolone level greater than 25 μg/L.

Within the follow-up period, 21 individuals with 8-h prednisolone levels greater than 25 μg/L successfully had their prednisolone dose decreased and remained well for at least 12 months following dose reduction. Early morning cortisol levels were also checked prior to prednisolone administration. Eight individuals were unexpectedly found to have recovery of the hypothalamic–pituitary–adrenal (HPA) axis during the follow-up period after being switched from hydrocortisone to prednisolone. Endogenous cortisol production only became apparent when they were switched to prednisolone. These individuals had a diagnosis of SAI and showed evidence of recovery of cortisol production during prednisolone weaning. Individuals were successfully weaned off prednisolone completely during the observed follow-up period if they had baseline cortisol levels of over 200 nM before prednisolone (Table 6) (18). Prior to their conversion to prednisolone, it is likely that five of these patients had supra-physiological glucocorticoid exposure. Four of these were iatrogenic from exogenous glucocorticoid exposure and one due to endogenous cortisol secretion due to Cushing’s disease. The remaining three patients had pituitary adenomas, and recovery of their HPA axis may have been due to a reduction in effective glucocorticoid exposure when they were switched from hydrocortisone to prednisolone. However, in PAI, prednisolone levels were used to optimise replacement prednisolone doses. Three patients required up-titration of their fludrocortisone dose. All three patients were clinically and biochemically stable on prednisolone for at least 1 year prior to acute up-titration of their fludrocortisone. In all three cases, fludrocortisone was increased based on electrolyte levels and clinical assessment during routine follow-up.

**Table 6:** Pathology of individuals successfully weaned off prednisolone during follow-up.

Cause of AI	Pathology	Patients successfully weaned off prednisolone (*n* = 8)
SAI	Unilateral adrenalectomy	
	Cushing’s syndrome	12.5% (*n=*1)
	Pheochromocytoma (ACTH co-secretion)	12.5% (*n* = 1)
SAI	Cushing’s disease	12.5% (*n=*1)
SAI	Pituitary adenoma	37.5% (*n*= 3)
TAI	Inflammatory bowel disease (glucocorticoid-induced adrenal insufficiency)	25% (*n* = 2)

SAI, secondary adrenal insufficiency; TAI, tertiary adrenal insufficiency.

## Discussion

This is the largest dataset of prednisolone pharmacokinetics in patients with hypoadrenalism and demonstrates that once-daily very-low-dose prednisolone is a safe and effective glucocorticoid replacement therapy. Dose titration remains a significant clinical challenge. Serum prednisolone levels can be used to optimise doses to ensure individuals receive the minimal effective dose and avoid excess steroid exposure. Dose decreases correlate well with clinical symptomatology. We have found a strong correlation between 4- and 6-h prednisolone level measurements compared with 8-h levels. This data support single time point blood sampling at either 4, 6, or 8 h to accurately gauge adequate replacement status. This offers flexibility and greater convenience for patients and clinicians in the outpatient setting.

For many years, individuals with AI have received over-replacement with oral glucocorticoids (19, 20). With higher doses of prednisolone, symptoms may be controlled, and individuals have a lower risk of adrenal crises. With prolonged excess steroid exposure, however small the excess, there is an increased risk of detrimental metabolic and bone side effects (6). Historically, 5–7.5 mg of prednisolone was a commonly used maintenance dose (21, 22, 23). However, this has been shown to be associated with increased morbidity, mortality, and healthcare costs (23).

By using prednisolone day curves to gauge the adequacy of replacement, maintenance doses can be reduced to a median replacement dose of 4 mg, as demonstrated in our patient population.

Patients with AI are known to have very low cortisol levels upon awakening, possibly explaining early morning fatigue and nausea. We therefore recommend patients take prednisolone as early as possible upon wakening. Other studies are trialling glucocorticoid pump replacement therapy to mimic circadian rhythms; however, there is limited evidence for their use so far (24, 25). The importance of pulsatile therapies in patients with Addison’s disease will be clearer once the Medical Research Council ‘PULSES’ trial is complete (MR/R010919/1).

Patient-reported symptoms and the clinician’s discretion are the main deciding factors on weaning regimens. Weaning may be challenging due to the adverse effects of glucocorticoid withdrawal (26). Prednisolone levels may offer an additional tool in this joint decision-making process and provide an evidence base to attempt prednisolone weaning in those who show signs of HPA axis recovery.

Our study is limited by the retrospective nature of data collection and accurate documentation. There is a potential for selection bias in the cohort of patients in whom a clinical decision was made to perform a day curve. However, doses can be tailored using a reliable assay, with a recommended target 8-h prednisolone trough value of 15–25 µg/L. We are the first centre to be using low-dose prednisolone in conjunction with serum prednisolone levels. In the absence of end-organ markers, these levels have been defined clinically (13, 15). Measuring prednisolone at 8 h may be inconvenient in an outpatient setting. Therefore, we have derived 4- and 6-h prednisolone target ranges. This will enable individuals to take their prednisolone dose in the morning to mimic the intrinsic circadian rhythm and enable earlier sampling. Prednisolone levels at 1 and 2 h are dominated by variability in absorption, show poor correlation with 8-h levels and, therefore should not be relied upon.

Prednisolone and prednisone are active and inactive glucocorticoids, respectively, and prednisone requires activation by hepatic first-pass metabolism by 11β-hydroxysteroid dehydrogenase. The bioavailability profiles of prednisone and prednisolone were similar, and there was no difference as indicated by the AUC comparisons and serum concentration–time curves.

The median elimination half-life was 3.1 h, which is comparable to data reported in other studies (17, 27). In countries where prednisolone is not available, an identical dose of prednisone can be used. This should not be confused with methylprednisolone, which is 20% more potent than both prednisolone and prednisone. It is important to note therefore that 4 mg methylprednisolone is equivalent to 5 mg of prednisolone and 5 mg prednisone (28).

Patients with autoimmune diseases are frequently managed with anti-inflammatory high-dose prednisolone. Following the remission of their primary disease, a large cohort is unable to stop glucocorticoids because of tertiary AI (glucocorticoid-induced adrenal suppression). The clinical implications of this reverberate across a wide range of specialities, including respiratory and rheumatology (23, 29, 30).

## Conclusion

Optimising prednisolone doses using prednisolone levels facilitates the reduction in the dose required in patients with AI, thereby reducing unnecessary glucocorticoid exposure and the associated side effects. Previous replacement doses of 5–7.5 mg are harmful. Patients should therefore be titrated to the lowest possible safe dose, and the majority of patients require 2–4 mg. Although glucocorticoids may have differential glucocorticoid receptor binding and downstream effects (24, 25), data from head-to-head trials including PRED-AID (NCT03936517) and HYPER-AID (NCT03608943) will provide further evidence for the use of low-dose prednisolone in AI.

There is inter-individual variability in prednisolone metabolism on a single given dose. For the vast majority within our cohort, prednisolone doses of 5 mg may be supra-therapeutic. The use of prednisolone levels will enable dose reduction and avoid adverse effects associated with excess glucocorticoid use. We have demonstrated a strong correlation between 8-h vs 6- and 4-h prednisolone levels. Sampling at earlier time points allows for greater flexibility for patients and clinicians, enabling optimum prednisolone titration.

## Declaration of interest

No conflict of interest.

## Funding

TT is funded by the NIHR, NIHR BRC and the Moulton Charitable Research Foundation. KM is funded by the NIHR BRC. SC is funded by a National Institute for Health Research http://dx.doi.org/10.13039/100005622 (NIHR), Doctoral Research Fellowship (Grant: DRF-2017-10-115) and the Imperial Health Charity http://dx.doi.org/10.13039/100013842.
